# MosChito rafts as effective and eco-friendly tool for the delivery of a *Bacillus thuringiensis*-based insecticide to *Aedes albopictus* larvae

**DOI:** 10.1038/s41598-023-29501-3

**Published:** 2023-02-21

**Authors:** Simone Pitton, Agata Negri, Giulia Pezzali, Marco Piazzoni, Silvia Locarno, Paolo Gabrieli, Roberto Quadri, Valentina Mastrantonio, Sandra Urbanelli, Daniele Porretta, Claudio Bandi, Sara Epis, Silvia Caccia

**Affiliations:** 1 Department of Biosciences, University of Milan, Milan, Italy; 2 Pediatric Clinical Research Center “Romeo ed Enrica Invernizzi”, University of Milan, Milan, Italy; 3 Italian Malaria Network, Inter University Center for Malaria Research, University of Milan, Milan, Italy; 4 CIMAINA, Department of Physics, University of Milan, Milan, Italy; 5 Fondazione UNIMI, Milan, Italy; 6 Present Address: Department of Biomedical, Surgical and Dental Sciences, University of Milan, Milan, Italy; 7 Department of Physics, University of Milan, Milan, Italy; 8 Department of Environmental Biology, Sapienza University of Rome, Rome, Italy

**Keywords:** Entomology, Microbiology

## Abstract

Adult mosquito females, through their bites, are responsible for the transmission of different zoonotic pathogens. Although adult control represents a pillar for the prevention of disease spread, larval control is also crucial. Herein we characterized the effectiveness of a suitable tool, named “MosChito raft”, for the aquatic delivery of a *Bacillus thuringiensis* var. *israelensis* (*Bti*) formulate, a bioinsecticide active by ingestion against mosquito larvae. MosChito raft is a floating tool composed by chitosan cross-linked with genipin in which a *Bti*-based formulate and an attractant have been included. MosChito rafts (i) resulted attractive for the larvae of the Asian tiger mosquito *Aedes albopictus*, (ii) induced larval mortality within a few hours of exposure and, more importantly, (iii) protected the *Bti*-based formulate, whose insecticidal activity was maintained for more than one month in comparison to the few days residual activity of the commercial product. The delivery method was effective in both laboratory and semi-field conditions, demonstrating that MosChito rafts may represent an original, eco-based and user-friendly solution for larval control in domestic and peri-domestic aquatic habitats such as saucers and artificial containers in residential or urban environments.

## Introduction

Mosquitoes (Diptera: Culicidae) are a major threat in public health since adult females are able to transmit parasites and pathogens to humans and animals during the blood meal^1–3^. In addition, globalization and climate change have loosened biogeographic barriers and species with high invasive potential have spread worldwide creating concerns about exotic vector-borne zoonoses outbreaks^2,4–6^. In this scenario, the Asian tiger mosquito *Aedes albopictus* (Skuse, 1894) (Diptera: Culicidae) represents a case point because records of appearance in novel habitats have increased exponentially in the last decades^7,8^. Indeed, indigenous to South-East Asia, islands of the Western Pacific and Indian Ocean, *Ae. albopictus* is now present worldwide^7,8^. *Ae. albopictus* females are aggressive biters throughout the day, and they are competent vector for at least 22 arboviruses^2,7–9^. Since its first appearance in Europe in 1979, this species has been implicated in dengue and chikungunya outbreaks and reasonable concern is rising about Zika emergence in Europe in the near future^2,4,10,11^. Thus, mosquito control definitely relieves the biting pressure by aggressive species but, most importantly, it represents the pillar of disease prevention. Therefore research efforts to develop novel effective and sustainable control strategies are strongly encouraged^4,7,12–14^.

To face the growing and global challenges in the control of vector-borne diseases, mosquito control must be tackled by Integrated Vector Management (IVM) that is a rational decision-making process consisting of a multi-level approach to optimize the use of different tools and strategies to make it efficient, cost effective, and sustainable^12,15,16^. Based on the constant engagement and mobilization of the communities, IVM includes vector surveillance and larval control that can significantly complement adulticiding in the mitigation of disease spread^15,17–20^. In particular, public education and community-based interventions for larval control are crucial in the case of highly anthropophilic and container-inhabiting species (e.g., *Ae. albopictus*) for which larval habitats are ephemeral, unpredictable and ubiquitous within domestic and peridomestic environments^11,12,21,22^.

When feasible, the primary intervention for larval source management is the reduction of the availability of larval habitats, e.g., avoiding stagnation of water by everyday observation and elimination of small water containers^4,11,12,19,21,22^. In addition, in Europe, several larvicides are available (a complete list can be found on the European CHemicals Agency website at https://echa.europa.eu/it/information-on-chemicals/biocidal-products) and their adoption is regulated by the legislative act EU 528/2012 on biocide registration and use, that aims to encourage the exploitation of products with low impact on human and animal health and on the environment^11,12^. Essentially, two product categories are available for mosquito larvae control in EU, namely insect growth regulators (IGRs, i.e., chitin synthase inhibitors and hormonal disruptors) and microbial bioinsecticides [i.e., formulates based on *Bacillus thuringiensis* var. *israelensis* (*Bti*) or on the combination *Bti*-*Lysinibacillus sphaericus* (*Ls*)]^11,12,21^. Although ascribed as chemicals, IGRs specifically target insect development and thus are relatively safe for non-target organisms with minor effects on aquatic insect fauna^11,23^. IGR-based formulations are important components in IVM since they are effective and long-lasting, especially diflubenzuron-based products. Nevertheless, resistance records have been described and the incidence of resistance should be taken into consideration when using these products extensively, for example planning the rotation of products with different active ingredients^4,12,24^.

On the other hand, microbial larvicides based on *Bti* and *Ls* are considered safe for the environment and very specific^25,26^; in particular, they are active by ingestion since the bacteria produce proteinaceous toxins that target the midgut epithelium of mosquito larvae^26–28^. Products based singly on *Bti* or *Ls* present advantages and drawbacks with respect to each other and to other insecticides. *Bti* (i) is scarcely persistent, especially in polluted and organically enriched water, and requires multiple applications^11,29^ however (ii) produces a blend of toxins (several Cry and Cyt toxins) and resistance outbreaks have never been registered, although a mild decrease in susceptibility and resistance to single toxins have been described^28,29^. Conversely, *Ls* (i) persists longer and recycle in the environment through infected larvae but (ii) insects are more prone to develop resistance to the binary toxin (Bin) responsible for its acute toxicity^26,30^. To avoid resistance spread no formulates with *Ls* alone are available on the market, whereas bioinsecticides based on both *Bti* and *Ls* have been developed to synergize the toxic effects of both and to partially compensate the lack of persistence of *Bti*. Nevertheless, combining *Bti* and *Ls* or *Bti* and IGRs (e.g., as in VectoPrime^®^) imposes unnecessarily a selection pressure by *Ls* and IGRs with a real chance of resistance alleles spread in mosquito populations. The use of these combinations would be avoided in the case of persistent *Bti*-based products, by making larval control more targeted and sustainable.

The present work aims to protect the benefits of the use of *Bti*-based bioinsecticides that suffer from lack of persistence but are highly recommended for their environmental sustainability and for their mode of action that prevent resistance development. We have recently developed a suitable delivery method for the oral administration of microorganisms or molecules to mosquito larvae using a chitosan-based hydrogel^31^. Herein the potential of this tool (i.e., “MosChito rafts”) for the targeted and long-lasting delivery of a *Bti*-based formulate to *Ae. albopictus* larvae was investigated.

## Results

### MosChito rafts attractiveness for *Ae. albopictus* larvae

**Figure 1:**
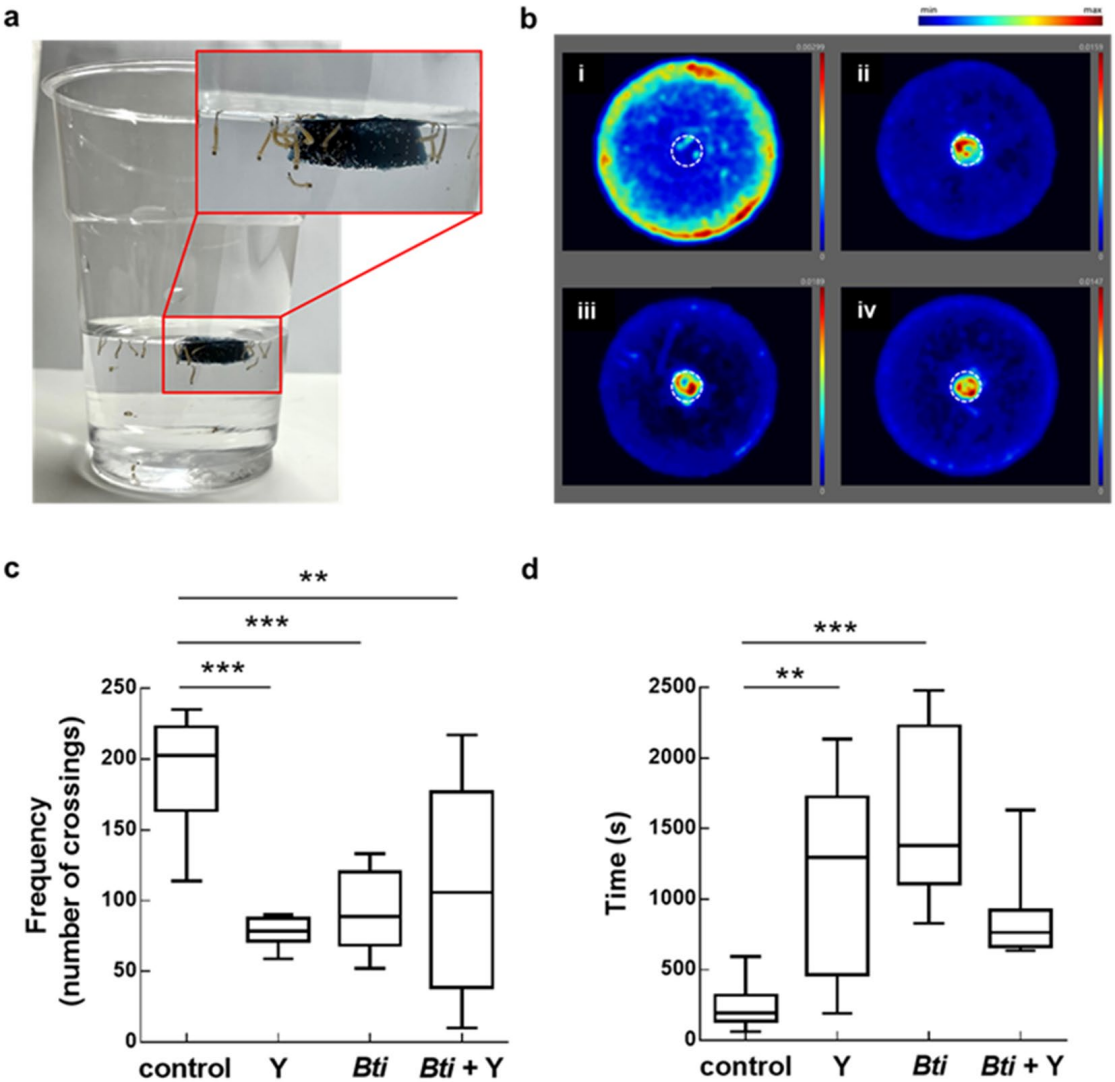
Exposure of *Ae. albopictus* larvae to MosChito rafts (**a**) and results of attractiveness assays (**b–d**). (**b**) Heatmaps of larval movement during the attraction experiment with control (i), Y (ii), *Bti* (iii), and *Bti* + Y (iv) rafts (see Methods for colour interpretation). In (**c,d**), crossing frequency of larvae in the zone at the border of the Petri dish and the cumulative duration of larvae permanence in the center zone are respectively represented. Data are reported as mean ± standard errors (**P < 0.01, ***P < 0.001).

In the present study we intended to validate floating hydrogel rafts consisting of chitosan crosslinked with genipin that have been recently developed^31^, for the delivery of a *Bti*-based formulate to control mosquito larvae with bioassays on *Ae. albopictus* larvae (Fig. 1a).

Larvae movement was measured and represented by cumulative heatmaps (Fig. 1b). In the case of control rafts (Fig. 1b, i), light blue-white halos were present in the whole test area except for the borders where yellow and red signals were intense, demonstrating that the larvae were inclined to move intensely and rest for long time at the borders, likely because control rafts were not attractive to them. In contrast, larvae exposed to Y, *Bti*, and *Bti* + Y (Fig. 1b, ii, iii, and iv respectively) rafts showed a tendency of staying around the raft itself, where a red-yellow halo is present, while the rest of the test area remained dark-blue coloured due to fewer movements and/or shorter permanence. In summary, Y, *Bti* and *Bti* + Y rafts attracted the larvae that perceived the presence of yeast, *Bti* or both in the rafts, and tended to stay close to them. These results were confirmed by a higher mean number of crossings of the larvae close to the border of the Petri dish in the case of control rafts compared to the other rafts (Fig. 1c) (F_(3, 35)_ = 9.548, *P* < 0.001, with *P* < 0.001 for control vs Y; *P* < 0.001 for control vs* Bti*, and *P* < 0.01 for control vs* Bti* + Y) and higher larvae permanence in the center zone in the case of Y and *Bti* compared to control (Fig. 1d) (F_(3, 29)_ = 8.495, *P* < 0.001 with *P* < 0.01 for control vs Y, *P* < 0.001 for control vs* Bti*). In the case of *Bti* + Y rafts no significant difference compared to controls was observed, although a tendency was present (*P* = 0.106) (Fig. 1d). Overall, contrary to previous reports, yeast did not act as a lure^32–35^ and attractiveness assays have shown that MosChito rafts attracted larvae per se, thus without the addition of yeast in the hydrogel.

### Insecticidal activity of MosChito rafts against *Ae. albopictus* larvae

**Figure 2:**
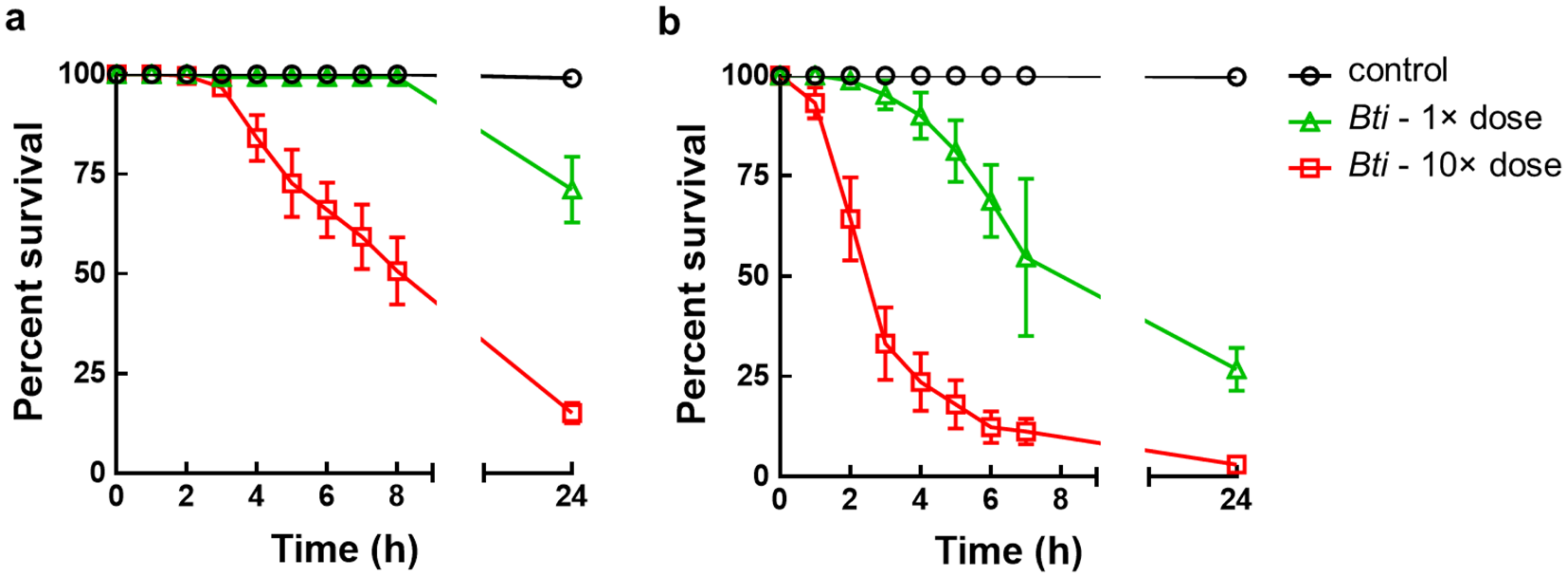
Bioassays with 3rd (**a**) or 4th (**b**) instar *Ae. albopictus* larvae exposed to control and *Bti* rafts. Two different doses of *Bti* were included in MosChito rafts (see Methods for the details) and survival of larvae was recorded at different times during 24 h. The values reported are the mean ± standard errors. Statistical significance of survival decrease in *Bti* exposed larvae compared to controls is reported along with the description of the results obtained.

Bioassays clearly showed that the insecticidal effect of MosChito rafts is (i) dose-dependent, and (ii) slower and lower on 3rd instar larvae compared to 4th instar larvae (Fig. 2). Indeed after 7 h, 3rd instar larvae exposed to the 1× dose were almost all alive, whereas about 50% of 4th instar larvae were dead (Fig. 2a,b). On the contrary, MosChito rafts with the higher dose were highly effective on both larval instars. Survival of 3rd and 4th instar larvae decreased significantly compared to the controls after 4 or 2 h of exposure to the higher dose respectively (for 3rd instar larvae F_(2, 21)_ = 11.49, *P* < 0.001; for 4th instar larvae F_(2, 33)_ = 10.13, *P* < 0.001) (Fig. 2a,b). After 24 h, MosChito rafts with the 10× dose killed more than 80% of 3rd and almost all 4th instar larvae (for 3rd instar larvae F_(2, 27)_ = 89.68, *P* < 0.001; for 4th instar larvae F_(2, 33)_ = 387.00, *P* < 0.001).

**Figure 3:**
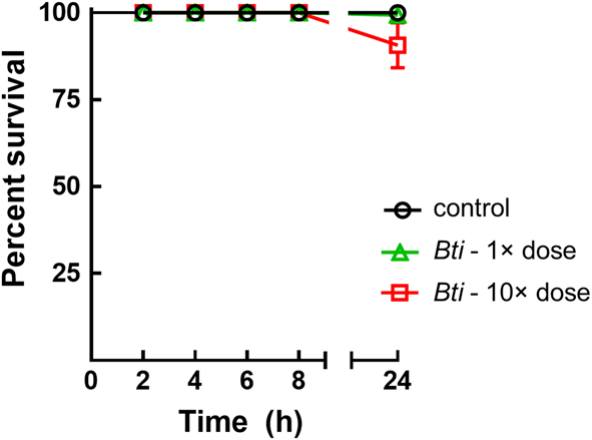
Bioassays with a mix of 3rd and 4th instar *Ae. albopictus* larvae to check whether *Bti* is released by MosChito rafts. Briefly, the rafts were left in 100 ml of tap water for 24 h and then the water was used to perform a 24 h time-course assay of survival with the larvae. The values reported are the mean ± standard error. The only statistically significant difference was observed at 24 h where 10× dose *Bti* induced a statistically significant, albeit small, survival decrease compared to other rafts (F_(2, 15)_ = 10.76, *P* = 0.0013, *P* < 0.01).

To unequivocally demonstrate that MosChito rafts are active by ingestion of the hydrogel, a bioassay was performed exposing larvae to the water in which the rafts were left 24 h and then removed (Fig. 3). The data clearly showed that there was no toxicity in the water itself after removing MosChito rafts and thus the toxicity reported in the insecticidal activity test was almost entirely due to the consumption and direct ingestion of the hydrogel with *Bti* by *Ae. albopictus* larvae (Fig. 3).

**Figure 4:**
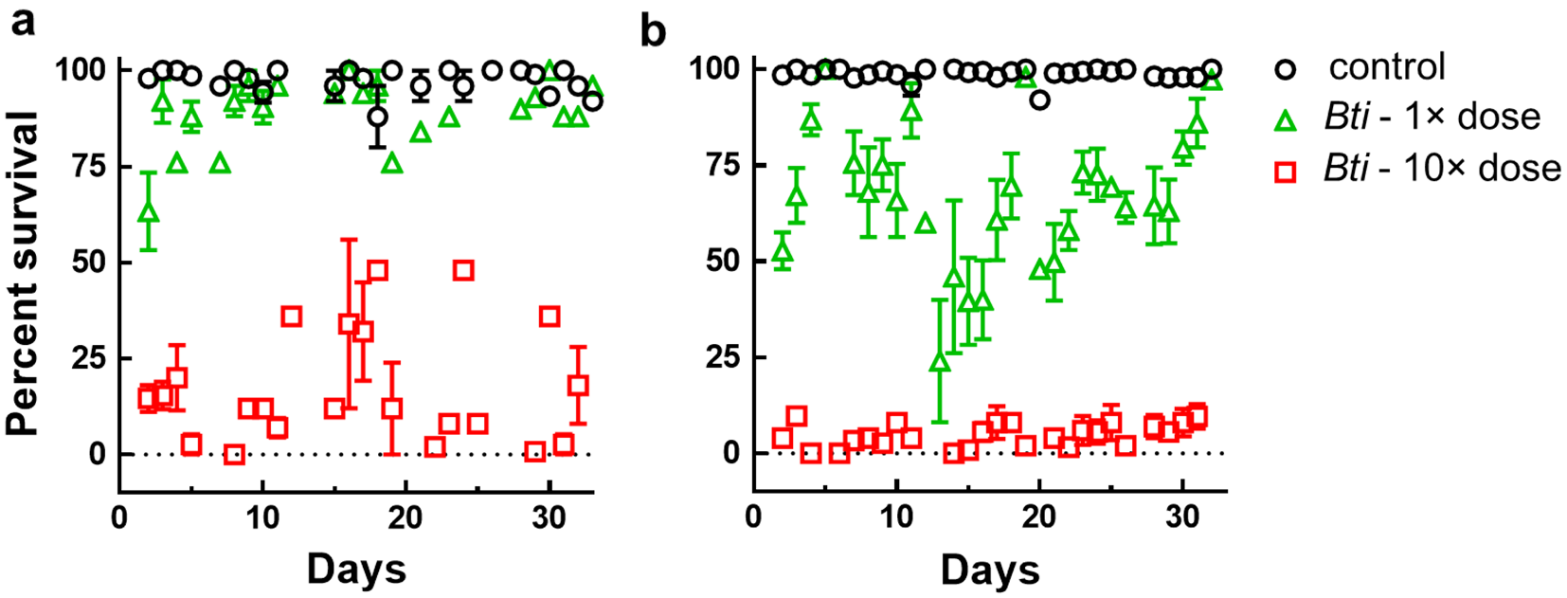
Bioassays with 3rd (**a**) or 4th (**b**) instar *Ae. albopictus* larvae exposed to control and MosChito rafts during long periods. Two different doses of *Bti* were included in the rafts (see Methods for the details) and 24 h survival of the larvae was recorded over a period of at least 30 days with the same rafts. The values reported are the mean ± standard errors. MosChito rafts toxicity was maintained during the test period (*P* < 0.001).

At the same time, data obtained during a month period demonstrated that MosChito rafts maintained unaltered toxicity against *Ae. albopictus* larvae over time for both tested doses of *Bti* (*P* < 0.001) (Fig. 4). It is worth mentioning that the slightly reduced toxicity in 3rd instar larvae (Figs. 2a, 4a) compared to 4th instar larvae (Figs. 2b, 4b) was likely due to the ingestion of lower quantities of hydrogel containing the *Bti* during 24 h of exposure.

**Figure 5:**
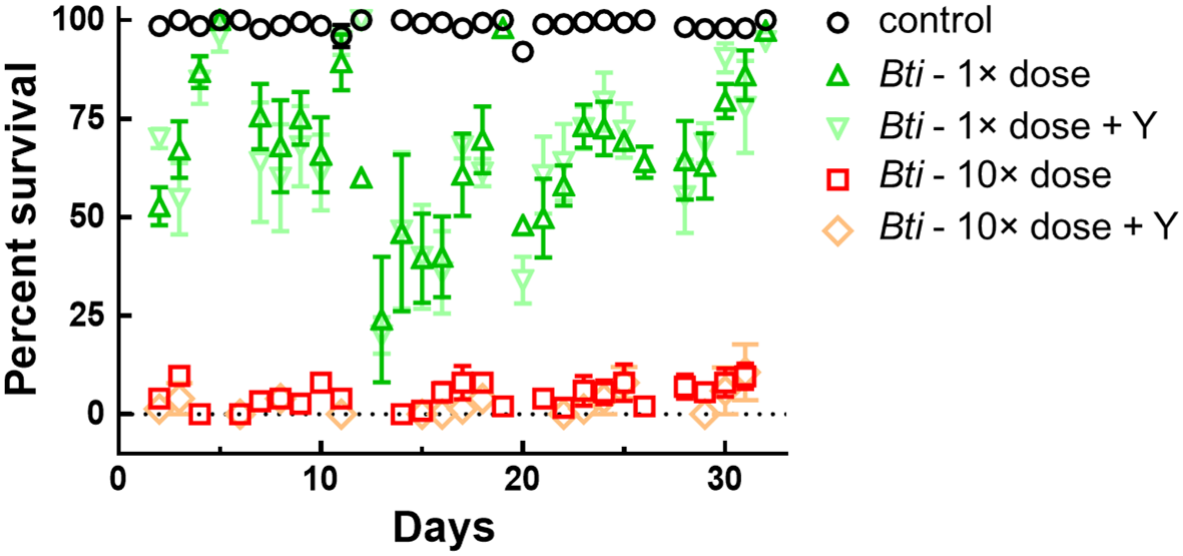
Bioassays with 4th instar *Ae. albopictus* larvae exposed to control, *Bti*, *Bti* + Y rafts (at two different doses, 1× and 10×). 24 h survival of larvae was recorded over a period of at least 30 days. The values reported are the mean ± standard errors. No statistically significant difference due to the presence of yeast between *Bti* and *Bti* + Y rafts was observed (*P* = 0.156).

Although MosChito rafts attractiveness assays (Fig. 1) did not show a significant difference in the movement of the larvae between *Bti* and *Bti* + Y rafts, a bioassay was performed to check whether the presence of yeast may have any effect on MosChito rafts toxicity, for instance by phagostimulating the larvae and thus boosting toxicity. This hypothesis was not supported by the results (Fig. 5), indeed the presence of yeast in the rafts with 1× dose *Bti* did not show increased toxicity expected if the presence of yeast would have induced a higher consumption of the rafts (*P* = 0.156). As expected, *Bti* and *Bti* + Y rafts with the higher dose caused similar mortality, closed to 100% (*P* = 0.156).

### Semi-field bioassays

**Figure 6:**
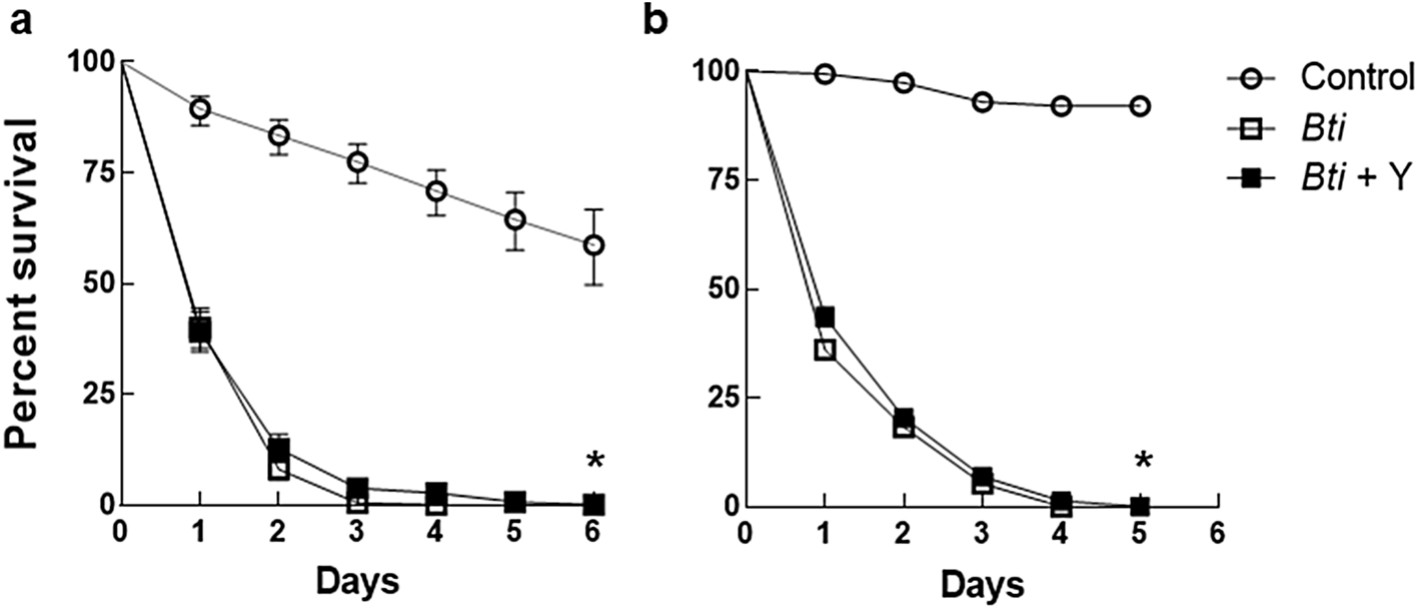
Bioassays in semi-field conditions with laboratory strains of *Ae. albopictus* established about 20 years ago (Rimini strain) or established less than 1 year prior to the experiments (Levate strain). Larvae of Rimini (**a**) or Levate (**b**) strain were exposed to control, *Bti* or *Bti* + Y rafts and survival was recorded every 24 h. The results are represented as mean ± standard error: curves that significantly differ from controls are indicated with an asterisk (*P* < 0.0001).

Efficacy of MosChito rafts in the natural context was then tasted in semi-field bioassays: both strains were highly susceptible to *Bti*-containing rafts since after 1 day of exposure less than 50% survival was observed and after 5 days the mortality increased to almost 100%, compared to the controls (Fig. 6). The results showed that *Ae. albopictus* larvae with a genetic background presumably similar to that of the wild populations were also highly susceptible to *Bti* and did not show any behavioural characteristic that may cause control failure (e.g., lack of attractiveness or erosion activity and ingestion of MosChito rafts in a natural environment), thus validating MosChito rafts as an effective control tool.

## Discussion

The control of *Ae. albopictus* mosquitoes is tricky because they can breed in almost any type of water-filled containers and dry-resistant eggs can survive over several months^12,36^. Nevertheless, their attitude to vector arboviruses compels the development of novel and sustainable control strategies. MosChito rafts represent an original, eco-based and user-friendly solution for larval control in aquatic habitats such as saucers and artificial containers in residential or urban environments to prevent the development of the immature stages. Control of floodwater mosquitoes is often performed by predictable, extensive and inundative treatments of wetlands or water bodies by professionals (e.g., by backpack sprayer or even helicopter). On the contrary, larval control of container breeding mosquitoes as *Ae. albopictus* or *Ae. aegypti* requires a localized and targeted treatment of breeding sites which can be better accomplished by hand-application of larvicides to specific containers in private or public urban contexts^11,12,22,36^.

MosChito rafts have been conceived with highly tested biomaterials. Chitosan, the major component of the hydrogel, is a renewable resource since it is produced by deacetylation of chitin, the second most widely occurring biopolymer in nature after cellulose^37,38^. Besides availability, chitosan is characterized by non-toxicity, biodegradability, and biocompatibility. It is widely used in food packaging, water and wastewater treatments, cosmetics, and agriculture to improve crops growth^39–41^. Chitosan also represents a valuable raw material for innovative biomedical applications, as carriers for a variety of drugs, bandages and wound dressing, tissue engineering, and in nerve reparation^38,41^. In addition, genipin was adopted in the hydrogel as chitosan cross-linker, since biobased cross-linkers of plant origin are safe, environmentally sustainable, and renewable^42–44^.

The insecticidal activity of MosChito rafts relies on the inclusion of a *Bti*-based formulation to the initial hydrogel. *Bti* is a safe and effective bioinsecticide targeting mosquito larvae and is implemented in current control programs all over the world, including Europe^11,26,36^. The major concern about *Bti* use is the low persistence in the environment, mainly due to UV light exposure and microbic degradation, and thus multiple applications are required^26,28,45^. Available products stuffed with *Bti* as Mosquito Dunks^®^ or Culinex^®^ tabs are designed to be more or less rapidly dissolved in water and thus *Bti* is immediately exposed to water pH and UV light after its release. In addition, the amount of released *Bti* is not adjusted according to larval instar or density but is instead released in very high amounts to guarantee vector control. MosChito rafts’ mechanism of delivery is completely different. First, MosChito rafts containing a *Bti* formulate (i.e., VectoBac^®^ 12AS) to be attractive to *Ae. albopictus* larvae even in the absence of a lure, to be highly effective during the 1-month testing period (while the commercial liquid formulate that was used to make MosChito rafts persists fully active only for a few days, as reported in VectoBac^®^ 12AS data sheet), and that toxicity is mediated by the erosion of the soft hydrogel by the larvae mouthparts followed by ingestion. This characteristic is extremely important since it avoids dispersion of the bioinsecticide that is protected by the hydrogel from environmental abiotic and biotic stressors. Importantly, MosChito rafts allow to overcome the need for the addition of other insecticides (as *Ls* or IGRs) in *Bti* formulates to prolong the insecticidal effectiveness, a practice that dangerously imposes a selective pressure on larvae which may evolve in resistance to *Ls* and IGRs.

The European directive No. 528/2012 on biocidal products regulation made *Bti* one of the few larvicides authorized for mosquito control. Notwithstanding the individuation of new bioinsecticides remains a core effort for the improvement of mosquito larvae control strategies, the protection of the benefits of *Bti* use and the optimization of its performances by developing new delivery methods and/or by combining this bioinsecticide with other control strategies are also key issues. Recent works have demonstrated that the effectiveness of a *B. thuringiensis* strain active on lepidopteran pests (*B. thuringiensis* var. *aizawaii*, *Bta*) is enhanced when target insects are immune impaired by RNAi-mediated silencing of genes involved in cellular immune responses^46,47^. In addition, this approach can be exploited in the field by co-administration of the *Bta*-based formulate with transformed bacteria or plants as delivery vectors for immune silencing dsRNAs^48,49^. Similarly, MosChito rafts could be used as vectors for *Bti* in association with dsRNA nanocarriers^50–52^ or dsRNA-expressing microorganisms, as mosquito larvae have proved to be susceptible to environmental RNAi vectored by microorganisms, including *S. cerevisiae*^34,53–56^. *S. cerevisiae* in MosChito rafts can therefore be exploited as expression and delivery system for interfering RNAs or for other molecules able to complement or synergize *Bti* formulate activity. Research efforts in this direction are ongoing in our laboratory.

Likewise, the potential of this device has yet to be assessed for other mosquito species. Indeed, the possibility to control mosquito species that often share breeding containers will expand its potential. For instance, in Italy, the overlapping ecological niche and seasonal activities of the populations of *Ae. albopictus* and *Culex pipiens*^57,58^, could play to our benefit for a targeted control of both species with a single product. Furthermore, the application of MosChito rafts could be fruitful and promising against the larvae of the species *Cx. pipiens* which normally develop in containers characterized by a larger volume (*Ae. albopictus*: < 5 L; *Cx. pipiens* (s.l.): > 5 L)^59^.

In conclusion the present work represents a significant proof of concept that sets the stage for the development of diverse and effective control strategies for mosquito larvae. Indeed, in principle, any bioinsecticide active by ingestion (e.g., formulates that combine both *Bti* and *Ls*) can be included and delivered, and suitably transformed *S. cerevisiae* cells can boost the bioinsecticide activity.

## Methods

### Mosquitoes, microorganisms and reagents

Bioassays were performed using larvae of the Asian tiger mosquito *Ae. albopictus*. The Rimini strain was established in 2004 from mosquitoes collected in Rimini, Italy^60^ and some egg clusters were transferred to the insectary of the Department of Biosciences (University of Milan). The Levate strain was recently established (September 2020) from larvae collected in Levate (Bergamo, Italy). Unless differently indicated, the Rimini strain was used for the experiments. The colonies were maintained in the insectary under standard rearing conditions (27 ± 1 °C, 65%–80% relative humidity, 12:12 h light/dark photoperiod). Both strains were fed with fish food (Tetra-fish, Melle) for all larval instars and with sucrose solution (10% w/v in distilled water) at the adult stage. Females were fed with animal blood to allow egg development. The eggs were stored dry in the insectary and used, by rehydration, no later than 2 months after the laying. For hatching, tap water and different hatching media (broths referred to as HM from now on) were tested, including the medium suggested in literature that include beef meat extracts (0.029 g Lab-Lemco powder, 0.14 g peptone, 0.14 g yeast extract, 0.14 g NaCl in 1 L of distilled water, HM3)^61–63^ and 2 media developed in our laboratory (0.14 g Bacto™ tryptone, 0.14 g yeast extract, 0.14 g NaCl in 1 L of distilled water, HM1; 0.14 g Primatone^®^ peptone, 0.14 g yeast extract, 0.14 g NaCl in 1 L of distilled water, HM2). The simplest and cheap medium HM1 was preferred as hatching solution, since no statistical differences were observed in the percentage of hatched larvae after 24 h compared to more complex media (i.e., 70% of egg hatching after 24 h, see Supplementary Fig. S1 online). This method allowed to optimally synchronize the larvae development which is important to perform bioassays with several conditions and replicates at the same time.

*S. cerevisiae* cells, strain SY2080, included into hydrogels (see “MosChito raft production”) were grown in generic yeast extract peptone dextrose (YPD) medium enriched with 2% w/v glucose as nutrient source and with chloramphenicol (1 µg/ml) added as antibiotic. Thirty ml of yeast culture were placed into a 50 ml tube and pelleted by centrifugation for 10 min at 3500×*g* at room temperature, and the supernatant was discarded. For heat inactivation the pellet was placed in a 70 °C water bath for 2 h (protocol modified from Mysore et al., 2017)^34^. A suspension of 10^7^ heat killed cells/ml in water was used for rafts production. The commercial *Bti*-based product used in our experiment is VectoBac^®^ 12AS (Sumitomo Chemicals Italia SRL, Valent Biosciences).

Unless differently indicated, all reagents were provided by Sigma-Aldrich, Italy.

### MosChito raft production

A detailed description of the formulation of floating hydrogel baits (rafts) and their properties was reported in Piazzoni et al*.*, 2022^31^. Briefly, “control” rafts were prepared by mixing 1 ml of chitosan solution (10 mg chitosan dissolved in 1 ml of a 1% v/v acetic acid solution and added with 10 µl of 20 mM sodium dodecyl sulphate in water) with 100 µl of 44 mM genipin solution in 10% ethyl alcohol. The other rafts used in the experiments were prepared by adding to the control rafts (i) SY2080 strain *S. cerevisiae* cells (50 µl of an aqueous suspension with 10^7^ cells/ml) (i.e., “Y” rafts), or (ii) the *Bti*-based insecticide VectoBac^®^ 12AS (5 µl or 50 µl of the liquid formulate for the rafts used in laboratory tests and 100 µl for semi-field tests) (i.e., “*Bti*” rafts), or (iii) both *S. cerevisiae* cells and VectoBac^®^ 12AS (50 µl with 10^7^ yeast cells/ml and 5 µl or 50 µl of the *Bti* formulate) (i.e., “*Bti* + Y” rafts). Then, air bubbles were injected using a syringe pump (KD Scientific, Thermo Fisher Scientific) to allow raft flotation in water; finally, they were placed in aluminum moulds (1.260 ml volume per well) to obtained rafts of discoidal shape of 1.6 cm (diameter) × 0.5 cm (thickness) (i.e., 1.2 ml of volume). The final concentration of VectoBac^®^ 12AS in “MosChito rafts” was thus 4.2 µl/ml (indicated as “1× dose”), 42 µl/ml (indicated as “10× dose”), or 420 µl/ml (in semi-field tests). Moulds were then incubated overnight in a ventilated oven at 37 °C. For mosquito attractiveness assays, the rafts were cut to obtain smaller ones.

### MosChito rafts attractiveness assays

To assess whether mosquito larvae were attracted or repelled by control, Y, *Bti*, or *Bti* + Y rafts, attractiveness assays were performed. The dose of VectoBac^®^ 12AS used in these *Bti*, and *Bti* + Y rafts was the 10× dose (i.e., 42 µl/ml). For each assay one raft (0.5 × 0.5 cm) was fixed with a needle in the center of a Petri dish (90 × 15 mm) containing 5 ml of tap water. To record larvae movement, Petri dishes were then placed in the DanioVision™ observation chamber (Noldus Inc., Wageningen, The Netherlands) with a plateholder filled with water to maintain the temperature at 27 °C. One single *Ae. albopictus* 3rd instar larva was tested for each recording. At least 10 larvae for each raft type (control, Y, *Bti* or *Bti* + Y) were tested. During each acquisition, lasting one hour, larvae movements to 3 pre-identified concentric areas in the Petri dish were recorded by automated video tracking (EthoVision XT^®^ software, Noldus Inc.). Starting from the dish center these areas are referred as “central zone”, 0.5 cm to 2 cm from the center of the dish, “border zone”, corresponding to the most external zone of the dish, near the border, with a 0.7 cm width.

The data acquired were used to generate heatmaps describing larval movements in different zones (the images offer an intuitive and unique view of the data, where the colour represents the relative time spent in a certain area (blue, low; red, high), averaged over all larvae of each experiment) and to establish the frequency of crossings or the duration of the permanence of the larva in a particular zone. Data were then processed with the GraphPad Prism (GraphPad Software Inc. version 8, San Diego, CA, USA).

### Laboratory bioassays

Third and 4th instar larvae were exposed separately to the rafts according to the guidelines for laboratory and field testing of mosquito larvicides^64^. Briefly, batches of 25 larvae were transferred by means of plastic Pasteur pipettes to disposable plastic cups containing 100 ml of tap water. Rafts (controls and, depending on the experiment, 1× dose or 10× dose *Bti*, 1× dose *Bti* + Y, or 10× dose *Bti* + Y) were thus gently introduced in the water and experimental cups were put in the insectary (27 ± 1 °C, 65–80% relative humidity, 12:12 h light/dark photoperiod). In the case of the time course analysis of MosChito rafts toxicity (Fig. 2), survival was recorded at different time points within the 24 h period of exposure. In order to check whether the insecticidal activity was exclusively due to the ingestion of the hydrogel containing *Bti* and/or the *Bti* that was released into the water in the cups, the MosChito rafts were left in 100 ml of tap water for 24 h and then the water alone was used to perform a time-course assay of survival with a mix of 3rd and 4th instar larvae (cumulative survival of 3rd and 4th instar larvae was recorded after 2, 4, 6, 8 and 24 h). The experiment was performed in duplicate with 3 cups for each condition (control, 1× dose *Bti* and 10× dose *Bti*) and with 25 larvae for each cup. To measure the insecticidal activity of the rafts during time, after each 24 h bioassay (i.e., larval survival was measured 24 h after the exposure to MosChito rafts), rafts were moved to a new plastic cup, with fresh tap water and 25 larvae, to start a new 24 h bioassay. The insecticidal activity of MosChito rafts was recorded across a 30 days-period. Three batches of 25 larvae were used to measure survival for each experimental condition and experiments were repeated with rafts obtained with at least 2 independent preparations.

### Semi-field bioassays

*Ae. albopictus* larvae were tested in bioassays under semi-field conditions to evaluate the larvicidal efficacy of control, Y, *Bti* and *Bti* + Y rafts over time. These experiments were performed in the backyard of the Department of Biosciences of the University of Milan, from June to September 2021. Fifty *Ae. albopictus* larvae (Rimini strain) at different developmental stage were added to plastic containers with 200 ml of rainwater plus pebbles, leaves and sand to mimic the peridomestic environment where these mosquitoes normally breed and larvae develop. Survival was recorded every 24 h, until all larvae died or until all control larvae were pupated. Each bioassay was performed in triplicate and repeated 3 times. The same bioassays were performed following the same protocol using Levate strain of *Ae. albopictus*, a strain that has been established in the laboratory less than one year before the bioassays.

### Statistical analysis

Data obtained in attractiveness assays were checked for normality using GraphPad Prism (GraphPad Software Inc. version 8) and statistical significance of differences was assessed with One-way ANOVA tests followed by Tukey’s multiple comparison post-hoc test. Insect survival in laboratory tests was analysed by one-way ANOVA followed by Tukey’s post-hoc test (Figs. 2 and 3) or by General Linear Model (GLM, performed with RStudio v2022.2.3.492, RStudio Team 2020)^65^ (Figs. 4 and 5). Data from semi-field bioassays were analysed by Log-rank (Mantel-Cox) test and the comparison between groups was adjusted with FDR (false discovery rate). If not differently stated, statistical analysis was performed using GraphPad Prism.

## Supplementary Information

Supplementary Figure S1.

## Data Availability

All data relevant to the study are included in the article or uploaded as supplementary information. In addition, the datasets used and/or analyzed during the current study are available from the corresponding author on reasonable request.
