# Supplementary material for: MosChito rafts as effective and eco-friendly tool for the delivery of a Bacillus thuringiensis-based insecticide to Aedes albopictus larvae
Source: Sci Rep. 2023 Feb 21;13:3041. doi: 10.1038/s41598-023-29501-3 (PMC9944263; doi:10.1038/s41598-023-29501-3)
Supplement: Supplementary file 1 — Supplementary Figure S1. [file 41598_2023_29501_MOESM1_ESM.docx]

**Supplementary Figure S1.** **Effect of tap water and different media on the percentage of hatching during 24 h and hatching beginning time (insert) of *Ae. albopictus* eggs.** Clusters of about 100 dehydrated eggs were selected and put in tap water or in different Hatching Media (HMs) (see Methods for detailed composition), and hatching was monitored during time. Percent hatching in water was relatively low compared to HMs. For all HM media the percent hatching differed from water starting from 6-7 hours and reached for all of them 70 % at 24 h. Insert shows that the mean time when the first hatch was recorded (defined as T_zero_) was 3 h for water and the different HMs (*P* = 0.9238, One-way ANOVA). The values reported are the mean ± standard error of about 20 replicates for each condition. One-way ANOVA followed by Tukey’s multiple comparison test was performed for each time point (statistics parameters are shown in the table on the right of the graph).
